# Structural and Biochemical Insights into the Reactivity of Thioredoxin h1 from *Chlamydomonas reinhardtii*

**DOI:** 10.3390/antiox8010010

**Published:** 2019-01-01

**Authors:** Christophe H. Marchand, Simona Fermani, Jacopo Rossi, Libero Gurrieri, Daniele Tedesco, Julien Henri, Francesca Sparla, Paolo Trost, Stéphane D. Lemaire, Mirko Zaffagnini

**Affiliations:** 1 Laboratoire de Biologie Moléculaire et Cellulaire des Eucaryotes, Institut de Biologie Physico-Chimique, Unité Mixte de Recherche 8226 CNRS Sorbonne Université, 13 rue Pierre et Marie Curie, 75005 Paris, France; christophe.marchand@ibpc.fr (C.H.M.); julien.henri@ibpc.fr (J.H.); stephane.lemaire@ibpc.fr (S.D.L.); 2 Department of Chemistry “Giacomo Ciamician”, University of Bologna, via Selmi 2, 40126 Bologna, Italy; 3 Laboratory of Molecular Plant Physiology, Department of Pharmacy and Biotechnology, University of Bologna, via Irnerio 42, 40126 Bologna, Italy; jacopo.rossi13@studio.unibo.it (J.R.); libero.gurrieri2@unibo.it (L.G.); francesca.sparla@unibo.it (F.S.); paolo.trost@unibo.it (P.T.); 4 Bio-Pharmaceutical Analysis Section (Bio-PhASe), Department of Pharmacy and Biotechnology, University of Bologna, via Belmeloro 6, 40126 Bologna, Italy; daniele.tedesco@unibo.it

**Keywords:** *Chlamydomonas reinhardtii*, cysteine alkylation, cysteine reactivity, MALDI-TOF mass spectrometry, thioredoxin, X-ray crystallography

## Abstract

Thioredoxins (TRXs) are major protein disulfide reductases of the cell. Their redox activity relies on a conserved Trp-Cys-(Gly/Pro)-Pro-Cys active site bearing two cysteine (Cys) residues that can be found either as free thiols (reduced TRXs) or linked together by a disulfide bond (oxidized TRXs) during the catalytic cycle. Their reactivity is crucial for TRX activity, and depends on the active site microenvironment. Here, we solved and compared the 3D structure of reduced and oxidized TRX h1 from *Chlamydomonas reinhardtii* (CrTRXh1). The three-dimensional structure was also determined for mutants of each active site Cys. Structural alignments of CrTRXh1 with other structurally solved plant TRXs showed a common spatial fold, despite the low sequence identity. Structural analyses of CrTRXh1 revealed that the protein adopts an identical conformation independently from its redox state. Treatment with iodoacetamide (IAM), a Cys alkylating agent, resulted in a rapid and pH-dependent inactivation of CrTRXh1. Starting from fully reduced CrTRXh1, we determined the acid dissociation constant (p*K*_a_) of each active site Cys by Matrix-assisted laser desorption/ionization-time of flight (MALDI-TOF) mass spectrometry analyses coupled to differential IAM-based alkylation. Based on the diversity of catalytic Cys deprotonation states, the mechanisms and structural features underlying disulfide redox activity are discussed.

## 1. Introduction

Thioredoxins (TRXs) are small oxidoreductases that contribute, in most living organisms, to the control of cellular redox homeostasis. Indeed, they reduce disulfide bonds on numerous proteins involved in several processes, including antioxidant defense mechanisms, photosynthetic carbon metabolism, and protein folding [1,2,3,4]. In addition, TRXs have been suggested to control two additional Cys-based post-translational modifications, namely S-glutathionylation and S-nitrosylation, by catalyzing the reduction of glutathione-mixed disulfides (–SSGs) and *S*-nitrosothiols (–SNOs), respectively [5,6,7,8,9,10,11]. The redox activity of TRXs relies on the presence of two Cys residues in the conserved Trp-Cys-(Gly/Pro)-Pro-Cys (WC(G/P)PC) active site motif. Regardless of the reducing activity in which TRXs are involved, the N-terminal active site Cys (CysN) is responsible for the first nucleophilic attack on oxidized targets. While the subsequent catalytic steps underlying TRX-dependent protein deglutathionylation and denitrosylation are yet undefined, the reduction of protein disulfides implies the formation of a mixed-disulfide bond between the CysN and a Cys on target protein. Following the formation of the TRX-target complex, the C-terminal active site Cys (CysC) of TRX performs a second nucleophilic attack on the TRX-target mixed-disulfide, yielding a reduced target and oxidized TRX [12,13,14].

Thiol groups are weak acids, and the reactivity of Cys residues depends on their acid dissociation constant (p*K_a_*) that controls thiol deprotonation state under physiological pH conditions [15]. Noteworthy, a deprotonated Cys (i.e., Cys thiolate, −S^−^) is much more reactive than its protonated counterpart (i.e., Cys thiol, −SH). In general, multiple factors related to the Cys microenvironment contribute to its reactivity. The proximity of charged residues (e.g., Arg, Lys, His, Asp), a hydrogen-bond network, and the N-terminal position in α-helix, contribute to increase Cys reactivity by lowering its p*K_a_* [15,16]. In 1997, Holmgren and colleagues established the importance of the active site microenvironment for TRX catalysis [17]. In this study, the mutation of charged residues (Asp26 and Lys57), which are located nearby the catalytic Cys thiols, caused a drastic decline of *Escherichia coli* TRX activity [17]. Whereas the role of the conserved Lys residue is still unclear, the Asp residue was suggested to be involved in the deprotonation of active site CysC in both *E. coli* TRX and *Chlamydomonas reinhardtii* (CrTRXh1) [18,19]. By contrast, in TRX from *S. aureus*, the Asp residue did not appear to be critical for CysC deprotonation [20]. To date, only p*K*_a_ values of the two catalytic Cys from non-plant TRXs have been determined (p*K*_a_ = 6.7−7.4 and >9.5 for CysN and CysC, respectively) [16]. These p*K*_a_ values are mainly determined by a combination of interactions with charged residues and the hydrogen-bonding network.

In photosynthetic eukaryotes, the TRX family counts 7 classes (f, h, m, o, x, y, and z) comprising multiple isoforms (9 and 21 in *Chlamydomonas reinhardtii* and *Arabidopsis thaliana*, respectively) localized in plastids, mitochondria, and cytoplasm [1,3,4,21]. The catalytic role of plant TRXs in thiol switching mechanisms (e.g., dithiol/disulfide exchange reactions) has been extensively investigated both in vitro and in vivo (for reviews, see [3,4]. In addition, proteomic-based approaches allowed the identification of hundreds of putative TRX targets in plants [22,23], and more than 1000 targets in *Chlamydomonas* [24], suggesting that TRXs can control multiple cellular pathways by regulating protein redox states in photosynthetic organisms. Although plant TRXs have been thoroughly characterized at the functional level, structural data are still limited to only 11 three-dimensional structures available so far [4]. This list includes TRX h1 and m from *Chlamydomonas reinhardtii* (CrTRXh1, [19]; CrTRXm, [25], TRX h1, o1, and o2 from *Arabidopsis thaliana* (AtTRXh1, [26]; AtTRXo1 and AtTRXo2, [27]), TRX h1 and h2 from *Hordeum vulgare* (HvTRXh1 and HvTRXh2, [28]), TRX h1 and h4 from *Populus trichocarpa* (PtTRXh1, [29]; PtTRX h4, [30]), and TRX f and m from *Spinacia oleracea* (SoTRXf and SoTRXm, [31]). In all cases, TRXs structures were solved in the oxidized form, except for plastidial SoTRXm, and cytoplasmic HvTRXh2 for which the 3D structure of the reduced forms is available.

In the present study, we deepen the structural features of CrTRXh1, providing the 3D structures of both the reduced form and variants in which catalytic cysteines were mutated. Extensive comparison of CrTRXh1 with structurally solved plant TRXs uncovers a conserved 3D folding despite the low sequence identity. Further analysis suggests that plant TRX classes exhibit peculiar structural features for substrate recognition. Structural alignments and circular dichroism (CD) analysis of reduced and oxidized CrTRXh1 reveal an identical folding independent on the redox state. The analysis of the catalytic site microenvironment from the crystal structure of reduced CrTRXh1 indicates that a hydrogen-bonding network is the major factor determining CysN reactivity. Through mass spectrometry (MS) analysis coupled to pH-dependent iodoacetamide (IAM)-based alkylation, we experimentally determined the p*K*_a_ of both active site cysteines. Based on the diversity of catalytic Cys deprotonation states, we discussed the mechanisms and structural features underlying disulfide redox activity of TRX.

## 2. Materials and Methods

### 2.1. Material and Enzymes

All reagents were purchased from Sigma-Aldrich (St Louis, Missouri, MO, USA) unless otherwise indicated. Production and purification of recombinant CrTRXh1 (wild-type, C36S and C39S mutants) and nicotinamide adenine dinucleotide phosphate reduced (NADPH)-thioredoxin reductase B from *Arabidopsis thaliana* (AtNTRB) were carried out as previously reported [32,33].

### 2.2. Enzymatic Assay

Activity of CrTRXh1 was assayed by following the reduction of 5,5’-dithiobis-2-nitrobenzoic acid (DTNB). The reaction mixture contained 50 mM Tris-HCl (pH 7.9), 1 mM ethylenediaminetetraacetic acid (EDTA), 0.2 mM DTNB, 0.22 μM AtNTRB, 0.2 mM NADPH, and recombinant CrTRXh1 in a final volume of 1 mL. The reaction was started by adding CrTRXh1 at indicated concentration, and the reduction of DTNB was monitored by following the Abs_412_ increase associated with the formation of TNB^−^ (molar extinction coefficient at 412 nm = 14,150 M^−1^ cm^−1^). The reaction rates were corrected with a reference rate without CrTRXh1.

### 2.3. Inactivation of CrTRXh1 by IAM-Dependent Alkylation Treatments

Before each treatment, CrTRXh1 (20 or 100 μM) was incubated for 15 min at room temperature in 40 mM ammonium bicarbonate buffer (pH 8.5) in the presence of 5-fold molar excess of dithiothreitol (DTT). After incubation, the reduced protein (20 μM) was treated with 0.05 or 0.1 mM IAM without removal of the excess of DTT. At the indicated time, an aliquot of the sample (20 μL) was withdrawn for the assay of enzyme activity. To determine the pH-dependence of the inactivation of CrTRXh1 by IAM, aliquots of the reduced protein sample (100 μM) were diluted 5-fold in 100 mM sodium citrate (pH 5.0 and pH 6.0) or in 100 mM Bis-Tris (pH 6.5 and 7.0) or in 100 mM Tris-HCl (pH 8.0), and incubated with 0.1 mM IAM. After 2 min incubation, an aliquot of the sample (20 μL) was withdrawn for the assay of enzyme activity.

### 2.4. pK_a_ Determination by Matrix-Assisted Laser Desorption/Ionization-Time of Flight (MALDI-TOF) MS

For reduction of active site cysteines, 230 µM CrTRXh1 was incubated beforehand in 50 mM phosphate buffer (pH 7.1) for at least one hour at 25 °C with 2 mM Tris (2-carboxyethyl) phosphine (TCEP), a chemical reductant that does not react with Cys alkylating agents like IAM. For the p*K*_a_ determination of CysN, 2.1 µL of pre-reduced CrTRXh1 was directly diluted in 6.9 µL of reaction buffer at the indicated pH, and 1 µL of 1 mM IAM was added to give an IAM/TRX ratio of 2. After incubation (105 seconds), the reaction was quenched by adding 90 µL of a saturated sinapinic acid matrix solution freshly prepared in 30% acetonitrile and 0.3% trifluoroacetic acid. For p*K*_a_ determination of the CysC, pre-reduced CrTRXh1 (200 µM) was incubated (2 min) in 40 mM phosphate buffer (pH 7.1) with 2-fold excess of IAM to allow complete monoalkylation. Subsequently, 2.4 µL of pre-alkylated CrTRXh1 was directly diluted in 6.6 µL of reaction buffer at the indicated pH and 1 µL of 3.85 mM IAM was added to give a final IAM/TRX ratio of 10. After 2 min incubation, the reaction was quenched as described above. Reaction buffers used consisted in 200 mM acetate/acetic acid buffer for pH between 3.5 and 5.5; 100 mM phosphate buffer for pH between 6.0 and 8.0; 100 mM HEPES-NaOH for pH between 7.5 and 8.5, and 100 mM glycine buffer for pH values above 8.5.

For MALDI-TOF analyses, 1.8 µL of quenched mixtures were spotted on a sample plate and dried under a gentle air stream. Spectra were acquired in positive linear mode with an Autoflex MALDI-TOF/TOF mass spectrometer using FlexAnalysis 3.3 software (Bruker Daltonics, Bremen, Germany) after calibration on mono- and di-charged ions of equine cytochrome c. Peak area of reduced, mono- and dialkylated forms of CrTRXh1 were determined using DataAnalysis software (Bruker Daltonics, Bremen, Germany).

### 2.5. Identification of the Reactive Cysteine within the Monoalkylated Form of CrTRXh1

The monoalkylated form of CrTRXh1 was generated at pH 7.1 as described above and, after incubation, 0.5 mM DTT was added to quench the excess of IAM and thus avoid further alkylation during trypsin digestion. Alkylated CrTRXh1 (0.6 µg) was placed in 20 mM ammonium bicarbonate buffer (pH 8.5) and digested in a final volume of 6 µL with 50 ng of trypsin Gold (Promega) for 6 h. One microliter of this mixture was mixed with a half-saturated solution of α-cyano-4-hydroxycinnamic acid prepared in 50% acetonitrile/0.3% trifluoroacetic acid and then spotted on a MALDI sample plate. Peptide mass fingerprints were acquired on a Performa Axima mass spectrometer (Shimadzu, Manchester, UK) in positive reflectron mode, whereas collision-induced dissociation MS/MS spectra were acquired after selection of the alkylated cysteine-containing peptide ion and collision with helium gas.

### 2.6. Circular Dichroism Analysis

Circular dichroism (CD) analysis was performed on red- and ox-CrTRXh1 at room temperature using a Jasco J-810 spectropolarimeter (Tokyo, Japan). Far- ultraviolet (UV) CD measurements were performed in the 250−195 nm spectral range, using a 0.5 mm path length (high quality quartz cell; Hellma, Milan, Italy), a 20 nm min^−1^ scanning speed, a 4 nm response, a 2 nm spectral bandwidth, and an accumulation cycle of 3; solvent-corrected CD spectra were converted to molar units per residue (∆ε_res_). Samples for CD analysis were prepared at a nominal concentration of 10 μM in 30 mM Tris-HCl buffer (pH 7.9). Reduced CrTRXh1 was obtained following 30 min incubation in the presence of a 10-fold molar excess of TCEP. The exact concentration was determined from the absorbance at 280 nm (1 cm path length) based on the theoretical molar absorption coefficients of 13,980 M^−1^ cm^−1^ and 14,105 M^−1^ cm^−1^ for red- and ox-CrTRXh1, respectively [34]. Reduced CrTRXh1 was obtained by incubation with a 10-fold molar excess of TCEP for 30 min.

### 2.7. Crystallization and Data Collection

Protein samples, i.e., red- and ox-CrTRXh1, C36S and C39S mutants, were prepared in 50 mM Tris-HCl (pH 7.9), 1 mM EDTA, and concentrated at 18 mg/mL in the presence of 5 mM TCEP except for the oxidized form. They were crystallized by the hanging drop vapor diffusion method at 293 K. The drop composed of 2 μL of protein solution and an equal volume of reservoir, and was equilibrated against 750 µL of reservoir solution. The reservoir solutions were elaborated starting from crystallization conditions reported in Menchise et al. [19]. Crystals of all protein samples grew after one week from various solutions containing 20% (*w/v*) polyethylene glycol (PEG) 8K, or 20% (*w/v*) PEG 10K, or 10% (*w/v*) PEG 8K, or 10% (*w/v*) PEG 10K as precipitant buffered with 0.1 M sodium cacodylate or MES (2-(*N*-morpholino)ethanesulfonic acid) at pH 6.5, or 0.1 M HEPES-NaOH at pH 7.5, or 0.1 M Tris-HCl at pH 8.5.

Crystals were mounted from the crystallization drop into cryoloops, briefly soaked in a solution containing 22% (*w/v*) PEG 8K or 20% (*w/v*) PEG 10K or 11% (*w/v*) PEG 8K, and 11% (*w/v*) PEG 10K plus 20% (*v/v*) PEG400 as cryoprotectant, then frozen in liquid nitrogen. Diffraction images for red-, ox-CrTRXh1, and C36S mutant were recorded at 100 K at the at the European Synchrotron Radiation Facility (Grenoble, France, beam line ID23-1) using a wavelength of 1.0 Å, a Δφ of 0.15° and a detector distance of 290.04 mm (red-CrTRXh1), 294.66 mm (ox-CrTRXh1), 122.66 mm (monomeric C36S mutant), and 189.10 mm (dimeric C36S mutant). Diffraction images for C39S mutant were collected at Elettra synchrotron radiation source (Trieste, Italy, beam line XRD1) at a wavelength of 1.0 Å, a Δφ of 0.3°, and a detector distance of 200.00 mm.

Diffraction data were analyzed with XDS [35] for data reduction, with POINTLESS [36] for space group determination, and with SCALA [36] for scaling and merging. Statistics of data collection are reported in Table 1.

### 2.8. Structure Solution and Refinement

The lower resolution crystal structure of oxidized CrTRXh1 reported by [19] was directly used for refinement in the case of crystals isomorphous with the chosen model (Table 1), i.e., red- and ox-CrTRXh1, and dimeric C36S and C39S mutants. The same structure was used as a model to solve the structure of monomeric C36S mutant crystal (Table 1) by molecular replacement, using the software MOLREP [38]. The structures were refined with REFMAC 5.8.0135 [39] by selecting 5% of reflections for R_free_ calculation, and manually rebuild with Coot [40]. Water molecules automatically added with Coot [40], were visually checked and kept into the model if the relative electron density value in the (2F_o_ − F_c_) map was higher than 1.0 σ, and if stabilized by hydrogen bonds with the protein. Structures were further refined with PHENIX.REFINE [41]. The statistics of the refinement are shown in Table 1. Figures showing structures were prepared using PyMOL (The PyMOL Molecular Graphics System, Schrödinger, LLC).

### 2.9. Data Availability

The coordinates of the structural models and the experimental data (structure factors) are deposited in the Protein Data Bank (PDB). The assigned accession codes are: 6Q46 for red-CrTRXh1, 6Q47 for ox-CrTRXh1, 6Q6U for C39S mutant, and 6Q6T and 6Q6V for monomeric and dimeric C36S mutant, respectively.

## 3. Results

### 3.1. Sequence and Structural Comparison of Plant TRXs

**Figure 1:**
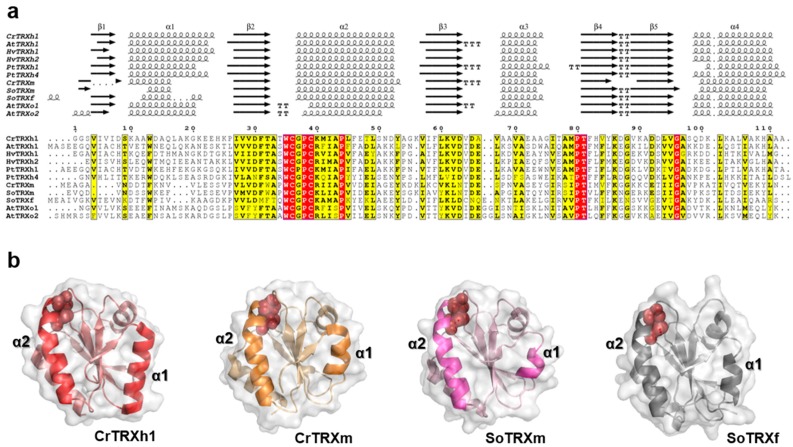
Sequence analysis and structural representation of plant TRXs. (**a**) Multiple sequence alignment of structurally solved plant TRX isoforms was performed with ESPript (http://espript.ibcp.fr, [42]) using *Chlamydomonas reinhardtii* (Cr) TRX h1 (Protein Data Bank (PDB) ID: 1EP7, [19]) and TRX m (PDB ID: 1DBY, [25]), *Arabidopsis thaliana* (At) TRX h1 (PDB ID: 1XFL, [26]), TRX o1 (PDB ID: 6G61, [27]), and TRX o2 (PDB ID: 6G62, [27], *Hordeum vulgare* (Hv) TRX h1 (PDB ID: 2VM1, [28]) and TRX h2 (PDB ID: 2IWT, [28]); *Populus trichocarpa* (Pt) TRX h1 (PDB ID: 1TI3, [29]) and TRX h4 (PDB ID: 3D21, [30]), and *Spinacia oleracea* (So) TRX f (PDB ID: 1F9M, [31]) and TRX m (PDB ID: 1FB0, [31]). Conserved residues are highlighted in white on red boxes, whereas residues with similar physicochemical properties are written in black on yellow boxes. The sequence identities were calculated with Clustal Omega [43]. (**b**) Ribbon representation of the three-dimensional structures of CrTRXh1, CrTRXm, SoTRXm, and SoTRXf. The structural elements α-helix 1 and α-helix 2 are labeled; and active site cysteines are shown as red balls.

Photosynthetic eukaryotes contain a large number of TRXs but, to date, only 11 isoforms were structurally characterized. These proteins belong to chloroplast f- and m-classes, cytoplasmic h-class, and mitochondrial o-class, and are from different photosynthetic organisms, such as the green alga *Chlamydomonas reinhardtii* (CrTRXh1 and m) and several land plants, such as *Arabidopsis* (AtTRXh1 and AtTRXo1/o2), barley (HvTRXh1/h2), poplar (PtTRXh1/h4), and spinach (SoTRXm and f). To achieve a better understanding of structure-function relationships among plant TRXs, we performed a primary sequence analysis coupled to structural alignment. As shown in Figure 1a, TRXs exhibit pairwise sequence identities ranging from ~21% to ~75%. The highest identity was observed between isoforms of mitochondrial o-class (AtTRXo1 and AtTRXo2), whereas the lowest identity was found between AtTRXo2 and SoTRXf. Among selected TRXs, sequence conservation is restricted to 9 residues, including the WC(G/P)PC active site motif, one stretch comprising two residues (Pro80-Thr81, numbered according to CrTRXh1), and two single amino acids (Pro44 and Gly96, numbered according to CrTRXh1) (Figure 1a).

To assess the structural similarity of plant TRXs, a 3D structure alignment was performed using PyMOL 2.1 and CrTRXh1 as template. This protein shares low identity with analyzed TRXs (31–53%) even compared with other h-class TRXs, PtTRXh1 being the closest homolog (Figure 1a). Despite the low sequence identity, the enzymes displayed highly similar 3D structures with a root mean square deviation (rmsd) ranging from 0.84 Å (CrTRXh1 versus HvTRXh1) to 2.06 Å (CrTRXh1 versus AtTRXo2). In-depth analysis of secondary structures revealed that the content of α-helices and β-sheets is also greatly conserved (Figure 1a). In CrTRXh1, around 45% and 26% of the total amino acids are involved in the formation of the α-helices and β-sheets, respectively. These values are substantially maintained in the other TRXs, with only the exception of SoTRXm and CrTRXm, which display a slight decrease of α-helix content (~35% and ~38%, respectively). This difference is mainly ascribed to the length of α-helix 1 (Figure 1a), which is composed by 4 residues in SoTRXm and 7 residues in CrTRXm and SoTRXf, while CrTRXh1 contains 14 residues (Figure 1a,b). All other h- and o-classes TRXs show, like CrTRXh1, a α-helix 1 of 11–14 residues (Figure 1a,b). The key structural element α-helix 2, which contains the active site motif WC(G/P)PC, was found to contain 16 residues in all TRXs except for CrTRXm (17 residues), and AtTRXo1/o2 (14 and 13 residues, respectively) (Figure 1a,b). The conservation of α-helix 2 is sensibly higher than the rest of TRX sequences, being in the ~39–81% range. Four amino acids are fully conserved, whereas the other residues, though not conserved, share common physicochemical properties (Figure 1a), suggesting their importance for proper folding of α-helix 2.

### 3.2. CrTRXh1 Shares the Substrate Recognition Loop with Other h-Class TRXs

**Figure 2:**
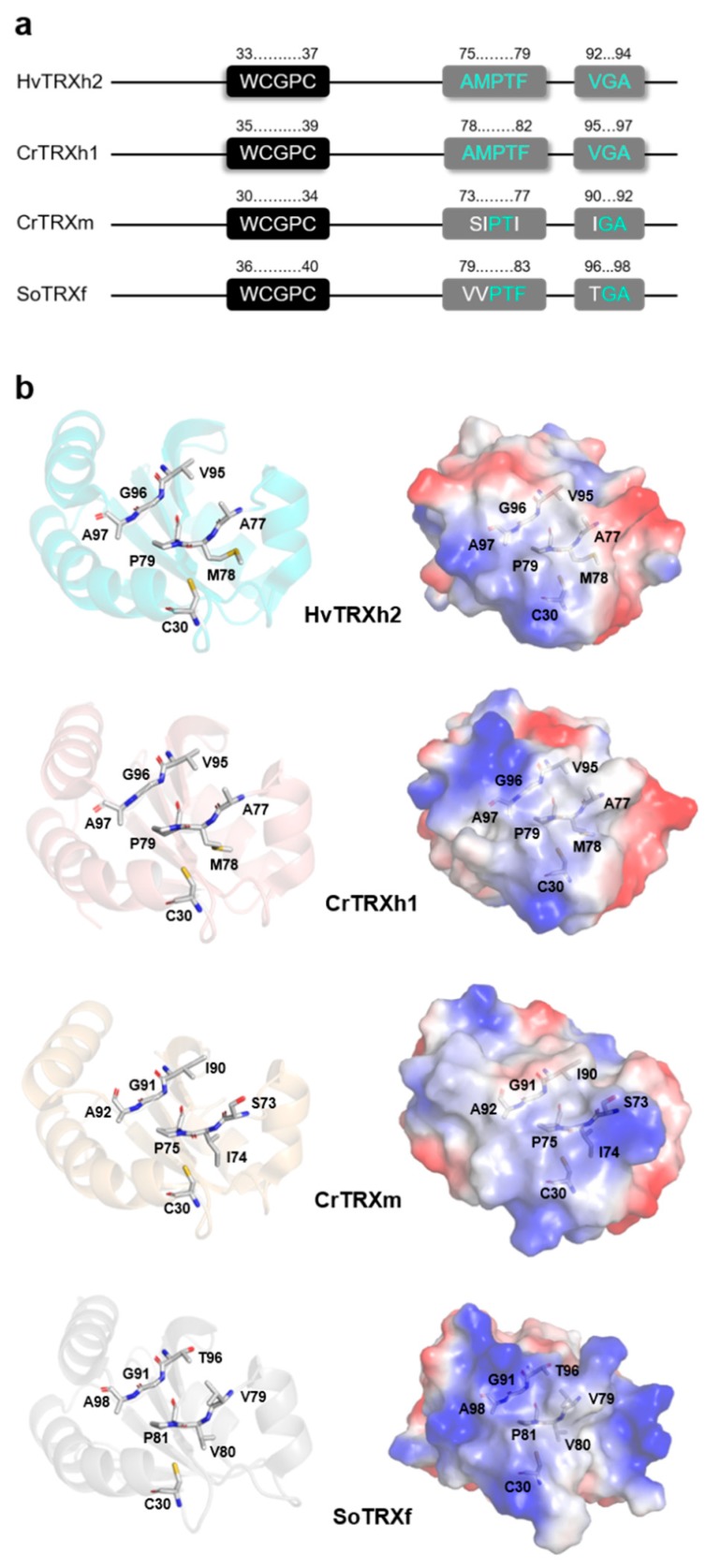
Sequence and structural analysis of the substrate recognition loop (SRL). (**a**) Schematic representation of the substrate recognition loop in HvTRXh2, CrTRXh1, CrTRXm, and SoTRXf. The active site motifs are highlighted in black, whereas the *cis*-Pro and Gly loops are highlighted in gray, with conserved and non-conserved residues indicated in cyan and white, respectively. The size of the strings is not proportional to the length in amino acids. (**b**) Ribbon representation of HvTRXh2, CrTRXh1, CrTRXm, and SoTRXf highlighting the residues forming the *cis*-Pro and Gly loops. (**c**) Electrostatic surface potentials were computed with the Adaptive Poisson-Boltzmann Solver (APBS) Electrostatics plugin in PyMOL (red for electronegative, white for neutral, blue for electropositive).

The global conservation of aligned plant TRX sequences is lower than 10%, a value that increases to 21% if sequences of h-class TRXs only are compared (Figure 1a). This difference is due to the presence of additional conserved residues randomly distributed along the sequences (Figure 1a), including two stretches composed by Ala–Met–Pro–Thr–Phe (AMPTF) and Val–Gly–Ala (VGA) (Figure 1a and Figure 2a).

These stretches include residues conserved in all TRXs (Pro–Thr and Gly, underlined, Figure 1a) but also other residues with little or no variations among h-class TRXs (Figure 2a and Figure S1). These stretches were recognized to be involved in target recognition by Maeda and colleagues [44]. In this study, the crystallographic structure of HvTRXh2 in complex with the target α-amylase/subtilisin inhibitor (BASI) was solved. In the HvTRXh2-BASI complex, the authors observed that the substrate recognition occurs through the WCGPC motif of HvTRXh2 along with residues Ala–Met–Pro (AMP, part of the conserved AMPTF sequence) and Val–Gly–Ala (VGA). The latter regions were named *cis*-Pro and Gly loops, respectively, and together with the active site motif form, the so-called substrate recognition loop (SRL). Interestingly, both *cis*-Pro and Gly loops are hydrophobic regions allowing van der Waals interactions and hydrogen-bonding with the backbone surrounding the target cysteine [45]. In their respective loops, the Pro supports correct positioning of Ala and Met, whereas the Gly confers flexibility to the flanking amino acids [44,45].

As shown in Figure 2a, CrTRXh1 shares with HvTRXh2 the SRL region, which is also partially conserved in other h-class TRXs (Figure S1). When we considered the other structurally solved TRXs (AtTRXo1, AtTRXo2, CrTRXm, SoTRXm, and SoTRXf), the aligned SRL region differs from h-class TRXs (Figure 2a and Figure S1). The major difference was observed in the Pro loop (AMP), in which Ala–Met are substituted by Ser–Ile and Val–Val in m-class TRXs and SoTRXf, respectively (Figure 2a,b, and Figure S1). A single substitution is observed in o-class TRXs, where a Val replaced the Met (Figure S1). By contrast, the Gly loop (VGA) only differs for the first residue Val that is replaced by Ile (m-class TRXs) or Thr (SoTRXf), or the last residue Ala substituted by a Val in AtTRXo2 (Figure 2a,b, and Figure S1). Taking into account the different residue substitutions, we can consider the replacement of hydrophobic residues (Ala and Val) with polar ones (Ser or Thr) as the most effective in modifying the electrostatic surface potentials (Figure 2b), that likely modulate the structural constraints involved in target recognition. Intriguingly, amino acid substitutions are comparable among TRX isoforms from the same class, suggesting that the SRL region could contribute to specifically guiding the recognition of protein targets by each TRX class.

### 3.3. Structure Analysis of Reduced and Oxidized CrTRXh1

**Figure 3:**
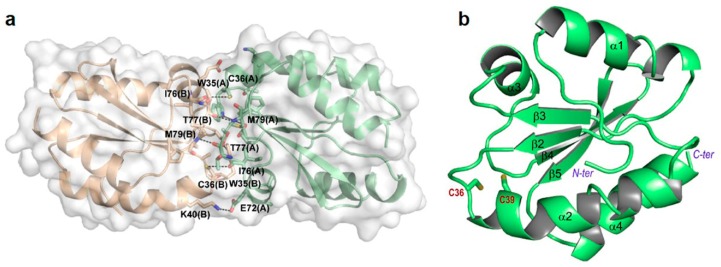
Crystal structure of red-CrTRXh1. (**a**) Cartoon representation of the asymmetric unit dimer. The dimer surface is shown in light gray. The interface interactions are shown, and the corresponding distances are indicated in Table S1. (**b**) Cartoon representation of the monomer structure. The secondary structure elements are shown, and the catalytic Cys36 (C36) and Cys39 (C39) are represented as sticks.

The structure of wild-type CrTRXh1 in its reduced and oxidized forms (red- and ox-CrTRXh1, respectively) were solved at a resolution of 1.70 and 1.57 Å, respectively. Crystals of red- and ox-CrTRXh1 are isomorphous to each other (Table 1), and to that previously reported for ox-CrTRXh1 structure (PDB ID: 1EP7, [19]). The asymmetric unit is composed by a non-covalent dimer, with the two monomers (chains A and B) related by a non-crystallographic two-fold axis. The total accessible surface area (ASA) is similar in the two enzyme forms (10,329 and 10,300 Å^2^ in red- and ox-CrTRXh1, respectively), while the buried surface area (BSA) is slightly lower in the reduced form (1117 Å^2^) with respect to the oxidized one (1169 Å^2^). Similarly, the calculated interface area between chains A and B is 556 Å^2^ in red-CrTRXh1 and 556 Å^2^ in ox-CrTRXh1. The interface is formed by 16 residues from each chain (Glu72–Met79, Ala33–Gly37, Val64–Asp65, and the single residue Lys40), and stabilized by two salt bridges, several hydrogen bonds, and hydrophobic interactions, which involve mainly Trp35 from both chains (Figure 3a) (Table S1).

The two independent chains A and B are very similar, and their superimposition gave an rmsd of 0.49 Å (on 112 aligned C_α_ atoms) and 0.48 Å (on 111 aligned C_α_ atoms) for red- and ox-CrTRXh1, respectively.

The CD spectra of red- and ox-CrTRXh1 are almost identical (Figure S2), suggesting a strong similarity in secondary structure between the two forms of CrTRXh1 in solution. In agreement with this observation, the protein redox state does not alter the overall protein folding in the crystal as well. Indeed, the superimposition of the dimers and the single monomers of red- and ox-CrTRXh1 resulted in rmsd values of 0.167 Å (on 222 C_α_ atoms) and 0.128 Å (on 111 C_α_ atoms), respectively. At structural level, CrTRXh1 folds in a 4-stranded β-sheet surrounded by 4 α-helices. The first β-strand, typically observed at the N-terminal end of plant TRXs (Figure 1a), is replaced by a long random coil region that precedes α-helix 1 (Figure 3b).

### 3.4. Active Site of Reduced and Oxidized CrTRXh1

**Figure 4:**
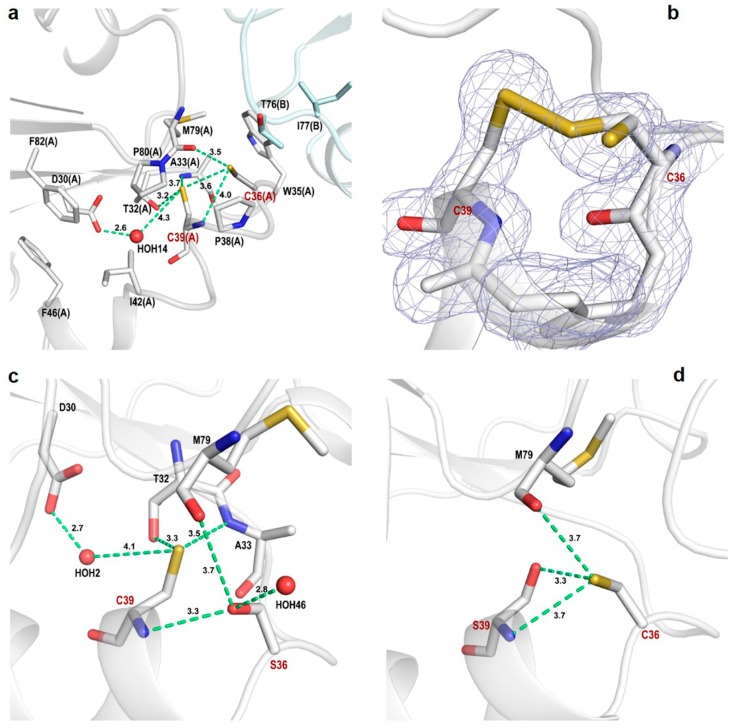
Active site of CrTRXh1. (**a**) Representation of red-CrTRXh1 active site. Residues are shown as sticks. The interactions of Cys36 (C36) and Cys39 (C39) are shown and the corresponding distances are indicated. (**b**) Representation of ox-CrTRXh1 active site and the corresponding 2F_o_ − F_c_ electron density map (contoured at 1.5 σ). The catalytic cysteines are represented as sticks. (**c**) Representation of C36S mutant active site. Residues are shown as sticks. The interactions of Ser36 (S36) and Cys39 (C39) are shown and the corresponding distances are indicated. (**d**) Representation of C39S mutant active site. Residues are shown as sticks. The interactions of Cys36 (C36) are shown and the corresponding distances are indicated.

The catalytic site Cys36 and Cys39 (corresponding to CysN and CysC, respectively) are located at the N-terminal end of α-helix 2 protruding from the protein surface of the monomer, but completely embedded in the interface if the crystallographic dimer is considered (Figure 3a). In the red-CrTRXh1 structure, cysteines are fully reduced, and the thiol groups stand at a distance of 3.6 Å (Figure 4a). By contrast, the 2F_o_ − F_c_ electron density map of ox-CrTRXh1 clearly shows a double conformation of Cys36 (Figure 4b), indicating that a portion of the molecules forming the crystal packing is not oxidized, even if the crystallization was performed under non-reducing conditions.

In red-CrTRXh1, Cys36 is the most exposed, showing an ASA of ~16.0 Å^2^, and its thiol group is hydrogen-bonded to the carbonyl group of Met79 (3.5 Å) and the thiol (3.6 Å), and the amide group (4.0 Å) of Cys39 (Figure 4a). If the crystallographic dimer is considered, the accessibility of Cys36 is drastically reduced (ASA = 1.8 Å^2^) by residues Ile76 and Thr77 of the adjacent protein chain interacting with the thiol group and contributing with Pro38, Met79, Pro80, and Trp35 to form a hydrophobic cavity (Figure 4a). Cys39 is buried in the active site cavity (ASA = 2.5 Å^2^) and surrounded by hydrophobic residues, such as Pro38, Ile42, Phe46, Met79, Pro80, and Pro82. However, the thiol group of this residue is involved in hydrogen bonds with Thr32, Ala33, Cys36, and the highly conserved Asp30 mediated by a water molecule (W14) (Figure 4a). This water molecule is structurally conserved in SoTRXm and SoTRXf [31], CrTRXf2 [46], and TRX from *E. coli* [47].

Taken together, these results indicate that both catalytic cysteines, though having different solvent exposure and surrounding microenvironment, form several conserved hydrogen bonds with neighboring residues. However, the deprotonation state of each catalytic Cys cannot be easily derived from structural observations and the real contribution of these interactions in the formation and stabilization of Cys thiolate(s) has to be experimentally determined.

### 3.5. C36S and C39S Mutants vs Wild-Type Reduced CrTRXh1

To gain further insights into the active site microenvironment, we structurally characterized Cys-to-Ser mutants of each catalytic Cys. Crystals of C36S (hereafter defined dimeric C36S) and C39S mutants were isomorphous to the wild-type protein crystal, and their 3D structures were solved at a resolution of 1.22 Å and 1.81 Å, respectively (Table 1). A new crystal form (orthorhombic, Table 1) was obtained, uniquely, for C36S mutant. This polymorph (hereafter defined monomeric C36S) diffracted at a maximum resolution of 0.94 Å, and it contains a single protein chain in the asymmetric unit. This result is quite unusual, since h-type crystal structures from different organisms, such as HvTRXh1 and h2 [28] and PtTRXh4 [30], show a non-covalent dimer in the asymmetric unit, except in the case of C61S mutant of PtTRXh4 [30]. Two chains in the asymmetric unit are also found in the crystal structure of other TRX isoforms from photosynthetic organisms, such as SoTRXf (short form; [31]), ox- and red-CrTRXm [31], and CrTRXf2, described in a companion paper of this journal issue [46], while one monomer is observed in the case of SoTRXf (long form; [31]) and cyanobacterial TRX 2 from *Anabaena* [48]. For the spinach enzyme, the different crystal packing was attributed to an inherent flexibility of its active site and to its interaction with the flexible N-terminal portion. Moreover, it has been proposed that in certain tissues where TRX concentration increases, the enzyme dimerization may have a regulatory role [49].

The different crystal packing does not affect the overall structure of C36S mutant. Indeed, the superimposition between dimeric and monomeric C36S give a rmsd ranging between 0.54 and 0.57 Å (on 110 aligned C_α_ atoms). A major difference is observed at the C-terminal part of α-helix 1 (residues 18–25) and in the linker region between α2 and β3 (residues 50–57), which are both involved in the interaction with symmetry-related molecules, as well as in the linker regions between β1 and α2 (residues 33–35), β3 and α3 (residues 63–70), and α4 and β4 (residues 77–80), all involved in the interface of the crystallographic dimer (Figure 3b). The comparison between red-CrTRXh1 and the mutants indicates higher differences in the case of C36S (rmsd values ranging from 0.49 and 0.55 Å for monomeric C36S, and from 0.15 and 0.69 Å for dimeric C36S) with respect to C39S (rmsd values ranging from 0.16 and 0.47 Å). These observations indicate that the mutation of Cys36 causes a higher perturbation of protein fold compared to Cys39 mutation. Main deviations are localized at the interface regions in the case of dimeric C36S, while at the C-terminal part of α-helix 1 and in the linker region between α2 and β3 in the case of monomeric C36S.The side chain of Ser36 is involved in several intramolecular hydrogen bonds with the carbonyl group of Met79 and the thiol and the amide group of Cys39, and it is further stabilized by a water molecule in the case of monomeric C36S (Figure 4c). Therefore, it appears that the mutation of Cys36 side chain with a more hydrophilic group perturbs the dimer interface favoring monomeric form.

Consistently with the minor structural alterations observed in Cys mutants compared to red-CrTRXh1, the position and the hydrogen-bond network of the unchanged catalytic Cys thiol groups are retained (Figure 4c,d).

### 3.6. CrTRXh1 Alkylation by Iodoacetamide is pH-Sensitive

**Figure 5:**
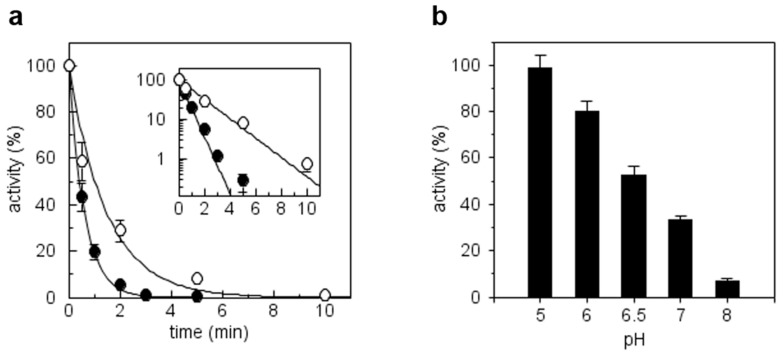
Alkylation treatment of CrTRXh1. (**a**) Reduced enzyme (20 μM) was incubated with 0.05 or 0.1 mM IAM (open and closed circles, respectively). Aliquots of the incubation mixtures were withdrawn at the indicated time points and the remaining TRX-dependent activity was determined. Data are reported as mean ± standard deviation (SD) (*n* = 3). When error bars are not visible, they are within the symbol. (**b**) The reduced enzyme was incubated at the indicated pH values in the presence of 0.1 mM. After 2 min incubation, an aliquot of the sample was withdrawn for the assay of enzyme activity. Data are reported as mean ± SD (*n* = 3).

In order to evaluate the presence of Cys thiolate(s) in reduced CrTRXh1, we first examined the sensitivity to iodoacetamide (IAM), a Cys alkylating agent that preferentially alkylates thiolates rather than protonated Cys [8,50]. As shown in Figure 5a, we observed a rapid and complete inhibition of TRX activity (i.e., NADPH/NTR/TRX-dependent DTNB reduction) after exposure to IAM used at 2.5- or 5-fold molar excess. A semi-logarithmic plot reveals that, in our conditions, inactivation kinetics were linear in the 0–3 min range (Figure 5a, inset).

To determine the pH-dependence of CrTRXh1 inactivation by IAM, we measured residual TRX activity following incubation with 5-fold molar excess IAM at different pH values. As expected, the inhibition of CrTRXh1 activity increased with increasing pH (i.e., increasing deprotonation of catalytic Cys) (Figure 5b). At pH 5, we observed no inhibition, while CrTRXh1 retained ~10% residual activity when alkylation occurred at pH 8 (Figure 5b). In the 6–7 pH range, the residual activity of CrTRXh1 progressively decreased, being around 50% at pH 6.5 (Figure 5b). Taken together, these results indicate that CrTRXh1 active site contains at least one Cys thiolate whose alkylation triggers protein inactivation.

### 3.7. pK_a_ Determination of CysN and CysC by Quantitative MALDI-TOF Mass Spectrometry

**Figure 6:**
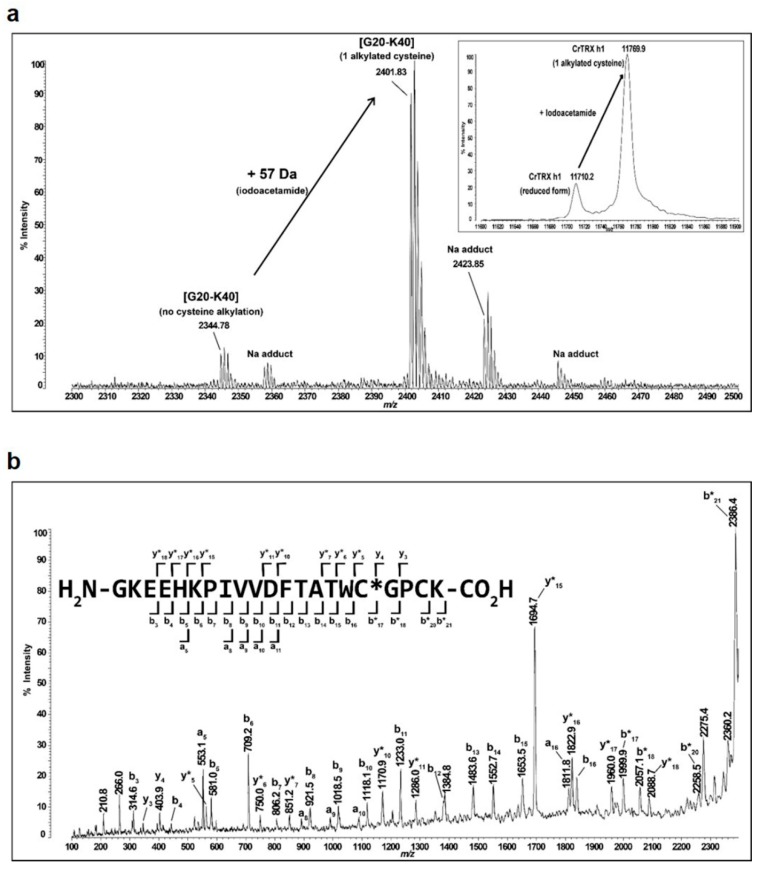
Mass spectrometry analysis of monoalkylated CrTRXh1. (**a**) Matrix-assisted laser desorption/ionization time-of-flight (MALDI-TOF) spectrum after tryptic digestion was determined for reduced CrTRXh1 after treatment with 2-fold molar excess of IAM at pH 7.1 (incubation time: 105 s). The active site containing peptide (Gly20–Lys40) is indicated (experimental mass of 2344.78 Da). The 57 Da shift after treatment with iodoacetamide (IAM) indicates monoalkylation of CrTRXh1 (experimental mass of 2401.83 Da). Inset, MALDI-TOF spectrum of intact protein was determined for reduced CrTRXh1 after treatment with IAM as described above. The experimental masses of 11,710.2 Da and 11,769.9 Da correspond to parental and monoalkylated enzyme, respectively. (**b**) Fragmentation spectrum of the monoalkylated peptide (Gly20–Lys40). The peptide ion (precursor mass at 2401.83 Da) was selected for fragmentation within the MALDI-TOF/TOF mass spectrometer by high-energy collision-induced dissociation using helium gas as collider. Fragment ions are annotated using the classical convention (y-ions when the charge is retained at the C-terminal side, b-ions when the charge is retained at the N-terminal side of the peptide sequence and a-ions correspond to a −28 Da carbonyl loss compared to b-ions). An asterisk (*) is used for fragment ions comprising the carbamidomethylated cysteine. The mass difference between y*_5_ and y_4_ ions, on the one hand, and b*_17_ and b_16_ ions, on the other hand, correspond exactly to 160 Da. This identifies unambiguously the CysN as carbamidomethylated.

MALDI-TOF mass spectrometry (MS) was already used to determine p*K*_a_ values of both active site cysteines of *E. coli* TRX [51]. Nevertheless, this method is based on MS analysis of tryptic-digested peptides after alkylation using the isotope-coded *N*-phenyl iodoacetamide. Recombinant CrTRXh1 contains only two cysteines and reaction of the fully reduced protein with IAM should increase its parental mass (11,712.6 Da) by 57 and 114 Da for single and double alkylation, respectively. Here, we employed MALDI-TOF MS coupled to IAM treatment to separate precisely at the protein level the parental and alkylated forms of CrTRXh1 with no further steps (Figure 6a).

In a first set of experiments, reduced CrTRXh1 was alkylated with a 10-fold excess of IAM at three different pH values (5.0, 7.0, and 9.0) (Figure S3). In agreement with activity assays (Figure 5), CrTRXh1 was almost insensitive to alkylation at pH 5.0 (Figure S3a). By contrast, at neutral pH, we observed that one active site cysteine was fully alkylated even for short incubation times (30 s), whereas the alkylation of the second residue appeared after 5 min, and progressively increased at a very slow rate (Figure S3b). Consistently, monoalkylation was extremely rapid at pH 9.0, and the peak corresponding to the doubly alkylated form started accumulating after 30 s, being around 80% and 100% after 5 and 20 min, respectively (Figure S3c). These preliminary data showed that CrTRXh1 could be entirely mono- or dialkylated, indicating that quantitative data for non-alkylated forms of CrTRXh1 were not biased by oxidized forms (i.e., non-reactive towards IAM). Moreover, these results indicate that the IAM/TRX ratio should be adapted to quantify, separately, the deprotonation state of the two active site cysteines. Considering that monoalkylation of CrTRXh1 was extremely rapid at both pH 7.0 and 9.0 in the presence of 10-fold excess of IAM, we evaluated the alkylation profile at IAM/TRX ratio of 2. Under these conditions, the occurrence of monoalkylated CrTRXh1 was linear within the 0–2 min range (Figure S4a,c).

The quantitative analyses of non-alkylated and alkylated forms (i.e., reduced and single or double alkylated species, respectively) at different IAM/TRX ratios were then exploited to determine the pH-dependence of CysN/CysC alkylation, and thus derive p*K*_a_ values. Before proceeding toward p*K*_a_ determination, we verified the Cys site in monoalkylated CrTRXh1 using MS and MS/MS analyses after trypsin digestion. As shown in Figure 6b, we demonstrated that the carbamidomethylated Cys in the monoalkylated form corresponded exclusively to CysN. By extension, we can firmly assume that alkylation of CysC occurs at higher IAM/TRX ratio correlating with the appearance of the dialkylated forms.

**Figure 7:**
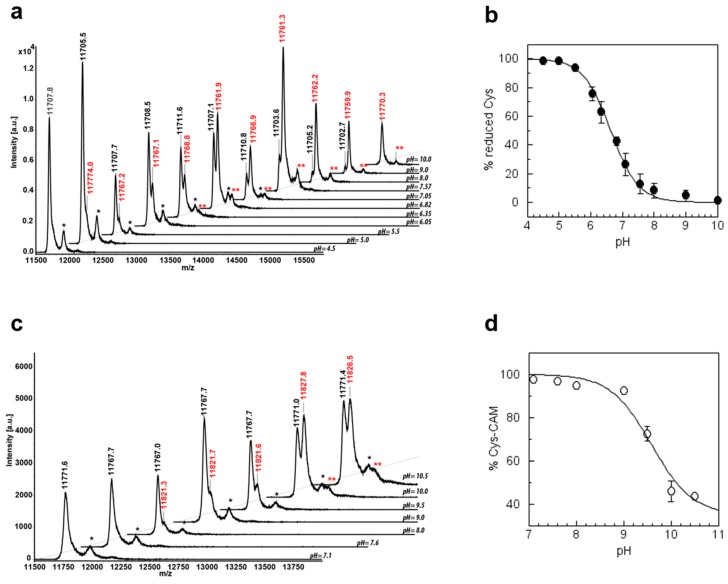
p*K*_a_ determination of active site cysteines. (**a**) Reduced CrTRXh1 was incubated with 2-fold excess of IAM at pH values ranging from 4.5 to 10 for 105 s before the reaction was quenched (see Material and Methods). Parental/monoalkylated (CrTRXh1-SH/CrTRXh1-CAM) protein ratios were determined by digital integration of MALDI-TOF mass spectrometry peaks labeled in black (CrTRXh1-SH) and red (CrTRXh1-CAM). The spectra are displayed as a function of pH. Matrix adducts for CrTRXh1-SH (one black asterisk) and CrTRXh1-CAM (two red asterisks) are indicated. (**b**) The fraction of reduced CrTRXh1 was measured by mass spectrometry as described in (**a**). The p*K*_a_ values was obtained by nonlinear regression using an adaptation of the Henderson–Hasselbalch equation [50]. Experimental data are displayed as mean ± SD (*n* = 3) and fitted to a full sigmoid. (**c**) Reduced CrTRXh1 was treated with 2-fold excess of IAM at pH 7 prior to incubation at pH values ranging from 4.5 to 10 for 2 min in the presence of 10-fold excess of IAM. After incubation, the reaction was quenched as in (**a**). Monoalkylated/dialkylated (CrTRXh1-CAM/CrTRXh1-CAM-CAM) protein ratios were determined by digital integration of MALDI-TOF mass spectrometry peaks labeled in black (CrTRXh1-CAM) and red (CrTRXh1-CAM-CAM). The spectra are represented as a function of pH. Matrix adducts for CrTRXh1-CAM (one black asterisk) and CrTRXh1-CAM-CAM (two red asterisks) are indicated. (**d**) The fraction of monoalkylated CrTRXh1 was measured by mass spectrometry as described in (**c**). The p*K*_a_ values were obtained by nonlinear regression as described above. Experimental data are displayed as mean ± SD (*n* = 2) and fitted to a full sigmoid.

In order to measure the reactivity of CysN, CrTRXh1 was incubated for 105 seconds at varying pH values using an IAM/TRX ratio of 2 (Figure 7a). By plotting the percentage of reduced CrTRXh1 (i.e., non-alkylated) against the pH, a sigmoidal titration curve was obtained allowing extrapolation of a p*K*_a_ value for CysN of 6.63 ± 0.13 (Figure 7b).

A similar procedure was exploited to evaluate the reactivity of CysC. The protein was first monoalkylated (2 min incubation at pH 7.0 with 2-fold excess of IAM), and then shifted to different pH buffers for additional 2 min in the presence of 10-fold excess of IAM (Figure 7c). The percentage of monoalkylated CrTRXh1 plotted against pH yielded a partial sigmoid titration curve from which a p*K*_a_ value of 9.53 ± 0.12 could be estimated for CysC (Figure 7d). Overall, these results indicate that CysN is mainly found in the nucleophilic thiolate form under physiological conditions, while CysC is fully protonated. The lack of intrinsic reactivity of CysC would suggest that the acquisition of a certain nucleophilicity could be related to conformational changes likely occurring during TRX–target complex formation.

## 4. Discussion

In all free-living organisms, TRXs play a conserved function that mainly consists of reducing protein disulfides. The catalytic activity of these enzymes depends on multiple factors strictly correlated to the structural features that control both the reactivity of active site cysteines and the specific recognition of target proteins. In order to get insight into structural-related catalytic features, we compared the 3D structures of plant TRXs and we observed an identical spatial folding, despite the low sequence conservation. This striking structural similarity is also accompanied by an almost equal content of secondary structures with only a few exceptions, mainly related to the length of α-helix 1. By focusing on the structural characterization of CrTRXh1, we observed that the folding of the protein remains almost unchanged between red- and ox-CrTRXh1, indicating that the protein conformation is not influenced by the redox state. An identical situation is also observed by comparing the 3D structures of red-CrTRXh1 and catalytic cysteine mutants. Nevertheless, the mutation of Cys36 alters the surface hydrophilicity of the catalytic site, hindering the formation of the non-covalent dimer always observed in crystals of wild-type CrTRXh1 and C39S mutant. This observation suggests that the catalytic CysN might be essential for the monomer–dimer equilibrium possibly involved in TRX regulation [49].

Based on the catalytic mechanism underlying TRX-dependent disulfide reduction, the reactivity of the Cys couple is crucial for TRX activity. Indeed, CysN performs a nucleophilic attack on the target disulfide forming a transient mixed disulfide with the target cysteine, whereas CysC acts as resolving Cys by reducing the TRX–target mixed-disulfide, yielding the reduced target and oxidized TRX. The deprotonation state of reactive Cys depends upon multiple factors, including the proximity of basic/acid residues, a hydrogen-bond network, and the dipole of α-helices [15]. In order to get insight into the microenvironment surrounding the catalytic cysteines, we analyzed the active site microenvironment of red-CrTRXh1. In the red-CrTRXh1, Cys36 is involved in three hydrogen bonds that are supposed to increase its reactivity. The p*K*_a_ value of Cys36 was experimentally determined to be ~6.6, using both activity assays and mass spectrometry analyses (Figure 5 and Figure 7). Therefore, we can assume that the deprotonation state strongly supports the ability of Cys36 to act as a thiolate nucleophile under physiological conditions, and that the hydrogen-bond network accounts for the low p*K*_a_ measured for Cys36 [16]. By contrast, we showed that Cys39 has p*K*_a_ value of ~9.5, though its thiol group is involved in four hydrogen bonds with neighboring residues. Based on these observations, we can question the assumption that the hydrogen-bond network is a major determinant of cysteine reactivity. Indeed, Cys39 constitutes an exception since the surrounding hydrophobic residues negatively affect the thiol proton abstraction typically favored by hydrogen bonds. Considering the unreactive nature of Cys39, we can hypothesize that its participation to the catalysis requires structural alterations of its microenvironment. Localized conformational changes, likely occurring after the formation of the TRX–target complex, could increase the reactivity of this residue [20,52,53].

Conservation of the three-dimensional fold and active site residues account for the functional redundancy of plant TRX family members. However, definite structural features are presumably designed to provide specificity towards target proteins. Mounting evidence supports that the specificity of TRXs towards target proteins relies on protein–protein interactions that are dependent on specific substrate recognition regions (e.g., SRLs) and complementary electrostatic surface potentials [46]. Consistently, we observed that each TRX has distinctive structural features that should guide the recognition of target proteins by facilitating protein–protein interactions. Despite the structural constraints involved in substrate recognition, we can assume that sequential catalytic steps ending with the nucleophilic attack of CysC to the TRX–target mixed disulfide control the catalytic activity of TRX. How the nucleophilicity (i.e., deprotonation) of this residue can be modulated by the formation of the binary complex between TRX and different protein partners is still an open question. A recent work by Messens and coworkers investigated, by molecular dynamics, the conformational changes occurring after the formation of TRX–target complex [20]. Although modest, the backbones of CysN and its preceding Trp slightly change and interact with the thiol of CysC with a final effect to decrease its p*K*_a_ by ~0.6 unit (from 8.9 to 8.3). Considering the crucial role of CysC in closing the catalytic cycle, the extent of deprotonation is thus fundamental to determine the overall catalytic efficiency of disulfide reductase activity of TRXs.

## 5. Conclusions

In conclusion, TRXs share an overall structural homology responsible for their oxidoreductase activity that primarily consists of controlling protein dithiol/disulfide exchange reactions. Despite this, each TRX isoform displays an accurate recognition of partner proteins that determines a regulatory control of specific metabolic pathways. Structural and biochemical features contribute to determine TRX specificity towards target proteins, and further studies are indeed required to shed light on the interconnected TRX features controlling the specific recognition of oxidized targets by one or multiple TRX isoforms and TRX-target interactions.

## Figures and Tables

**Table 1:** X-ray data collection and refinement statistics.

	Red-CrTRXh1	Ox-CrTRXh1	Monomeric C36S	Dimeric C36S	C39S
*Data collection*					
Unit cell (Å)	*a* = *b* = 48.77, *c* = 143.93	*a* = *b* = 48.68, *c* = 143.70	*a* = 60.78, *b* = 34.82, *c* = 48.16	*a* = *b* = 48.74, *c* = 143.19	*a* = *b* = 48.43, *c* = 143.66
Space group	P3_1_21	P3_1_21	P2_1_2_1_2	P3_1_21	P3_1_21
N° molecules ASU	2	2	1	2	2
Resolution range * (Å)	47.98–1.70 (1.76–1.70)	42.16–1.57 (1.63–1.57)	48.16–0.94 (0.96–0.94)	42.21–1.22 (1.24–1.22)	41.94–1.81 (1.84–1.81)
Unique reflections	22,494 (2165)	28,348 (2646)	64,505 (1579)	59,565 (2934)	18,676 (1044)
Completeness * (%)	99.3 (98.5)	99.5 (96.7)	96.5 (47.1)	99.7 (98.7)	99.6 (97.0)
R_merge_ *	0.145 (0.858)	0.072 (0.804)	0.043 (0.369)	0.063 (1.108)	0.111 (0.740)
R_pim_ *	0.064 (0.398)	0.034 (0.428)	0.033 (0.367)	0.028 (0.507)	0.057 (0.345)
CC_1/2_ *	0.980 (0.683)	0.998 (0.661)	0.997 (0.625)	0.998 (0.635)	0.990 (0.715)
I/(I) *	7.9 (2.2)	10.0 (1.5)	18.9 (1.7)	14.7 (1.7)	9.7 (1.9)
Multiplicity *	6.5 (6.6)	5.7 (4.9)	4.1 (1.6)	6.8 (6.6)	5.8 (5.7)
*Refinement*					
Resolution range * (Å)	42.24–1.70 (1.78–1.70)	42.16–1.57 (1.62–1.57)	48.16–0.94 (0.95–0.94)	42.21–1.22 (1.23–1.22)	41.94–1.81 (1.85–1.81)
Reflection used *	22,475 (2724)	28,277 (2671)	64,420 (1042)	59,506 (3597)	18,626 (2581)
R/R*_free_*	0.189/0.224	0.187/0.216	0.157/0.166	0.168/0.174	0.219/0.266
rmsd from ideality (Å, °)	0.006, 0.743	0.006, 0.820	0.006, 1.007	0.007, 1.079	0.002, 0.438
*N° atoms*					
Non-hydrogen atoms	1867	1832	1063	2015	1799
Protein atoms	1675	1660	856	1675	1637
Solvent molecules	192	172	207	346	162
Hydrogens	/	/	897	1724	/
*B* value (Å^2^)					
Mean	26.8	26.1	12.6	16.2	24.7
Wilson	25.8	23.6	8.4	12.6	24.0
Protein atoms	26.1	25.5	9.3	14.6	24.2
Solvent molecules	32.8	32.6	20.7	23.5	30.1
Heteroatoms	/	/	33.2	37.8	/
*Ramachandran plot* (%) ^§^					
Most favored	99.1	98.6	99.1	97.7	98.6
Allowed	0.9	1.4	0.9	2.3	1.4
Disallowed	0	0	0	0	0

* Values in parentheses refer to the last resolution shell; ^§^ As defined by MolProbity [37]. /: not determined.

## Supplementary Materials

The following are available online at http://www.mdpi.com/2076-3921/8/1/10/s1, Figure S1: Schematic representation of the Substrate Recognition Loop in AtTRXh1, HvTRXh1, PtTRXh1, PtTRXh4, AtTRXo1, AtTRXo2, and SoTRXm, Figure S2: Far-UV CD spectra of reduced and oxidized CrTRXh1 in Tris-HCl buffer (30 mM; pH 7.9), Figure S3: MALDI-TOF MS analysis of CrTRXh1 after IAM-dependent alkylation, Figure S4: Kinetic of alkylation of CysN and CysC from CrTRXh1, Table S1: Interface interactions including salt bridges and hydrogen bonds in the reduced and oxidized CrTRX h1 (cut-off distance 4.5 Å).

## Abbreviations

ASA	accessible surface area
BSA	buried surface area
CD	circular dichroism
Cys	cysteine(s)
DTNB	5,5’-dithiobis-2-nitrobenzoic acid
IAM	iodoacetamide
MALDI-TOF	matrix-assisted laser desorption/ionization-time of flight
MS	mass spectrometry
MS/MS	tandem mass spectrometry
SRL	substrate recognition loop
TCEP	Tris(2-carboxyethyl)phosphine
TRX	thioredoxin
