# Supplementary material for: Structural and Biochemical Insights into the Reactivity of Thioredoxin h1 from Chlamydomonas reinhardtii
Source: Antioxidants (Basel). 2019 Jan 1;8(1):10. doi: 10.3390/antiox8010010 (PMC6356897; doi:10.3390/antiox8010010)
Supplement: Supplementary file 1 [file antioxidants-08-00010-s001.pdf]

Supplementary Files:

Figure S1. Schematic representation of the Substrate Recognition Loop in AtTRXh1, HvTRXh1, PtTRXh1, PtTRXh4, AtTRXo1, AtTRXo2, and SoTRXm. The active site motifs are highlighted in black whereas the cis-Pro and Gly loops are highlighted in gray with conserved and non-conserved residues indicated in cyan and white, respectively. The size of the strings is not proportional to the length in amino acids.

Figure S2. Far-UV CD spectra of reduced and oxidized CrTRXh1 in Tris-HCl buffer (30 mM; pH 7.9).

Figure S3. MALDI-TOF MS analysis of CrTRXh1 after IAM-dependent alkylation. The pre-reduced CrTRXh1 was incubated with 10-fold excess of IAM at pH 5.0, 7.0, and 9.0. At indicated time, an aliquot was withdrawn and subjetced to MS analysis as indicated in «Material and Methods».

Figure S4. Kinetic of alkylation of CysN and CysC from CrTRXh1. The pre-reduced CrTRXh1 was incubated with 2-fold **(a)** or 10-fold **(b)** excess of IAM and analyzed by MS. Linear plots are indicative of the reduced (closed circles) or monoalkylated (open circles) state of the protein. Data are reported as mean of three replicates. **(c)** Logarithmic plot of data reported in panel (a) and (b).

Table S1. Interface interactions including salt bridges and hydrogen bonds in the reduced and oxidized CrTRX *h*1 (cut-off distance 4.5 Å).

Figure S1

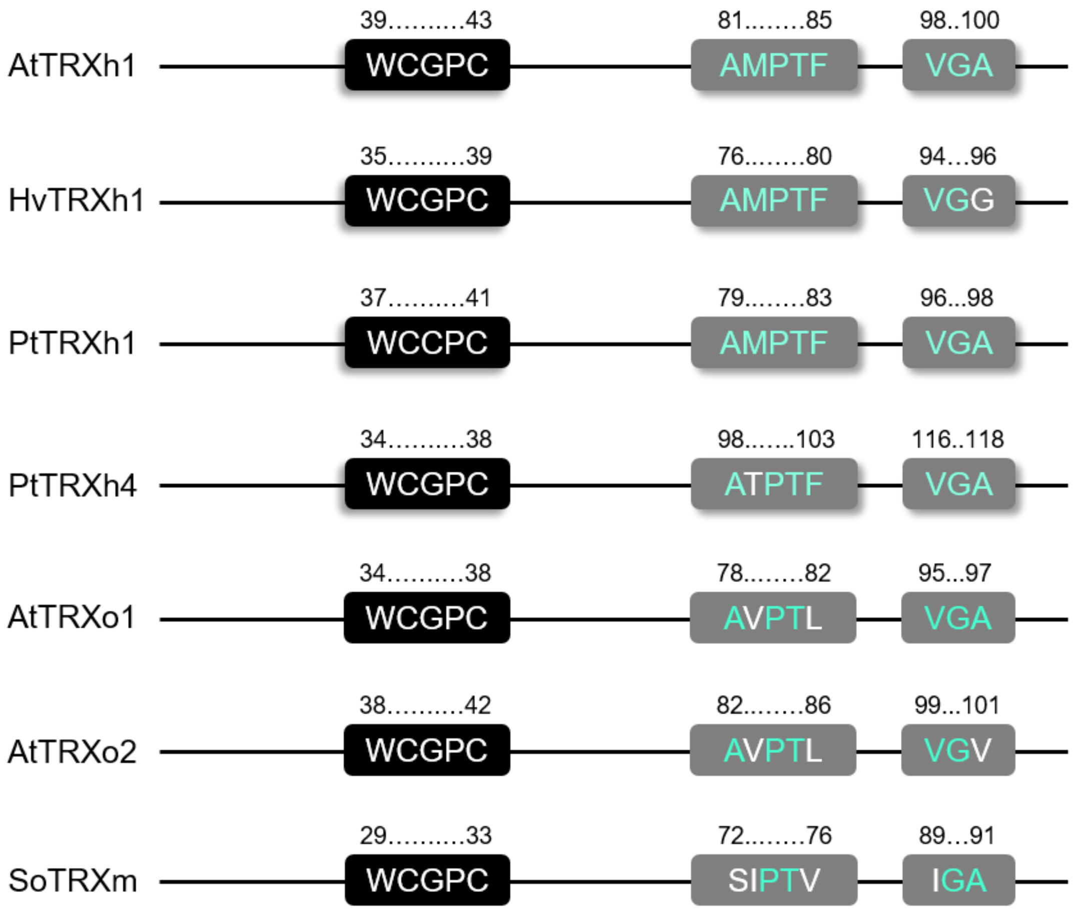

39.........43
81........85
98..100
AtTRXh1
WCGPC
AMPTF
VGA
35.........39
76........80
94...96
HvTRXh1
WCGPC
AMPTF
VGG
37.........41
79........83
96...98
PtTRXh1
WCCPC
AMPTF
VGA
34.........38
98.......103
116..118
PtTRXh4
WCGPC
ATPTF
VGA
34.........38
78........82
95...97
AtTRXo1
WCGPC
AVPTL
VGA
38.........42
82........86
99...101
AtTRXo2
WCGPC
AVPTL
VGV
29.........33
72........76
89...91
SoTRXm
WCGPC
SIPTV
IGA

Figure S2

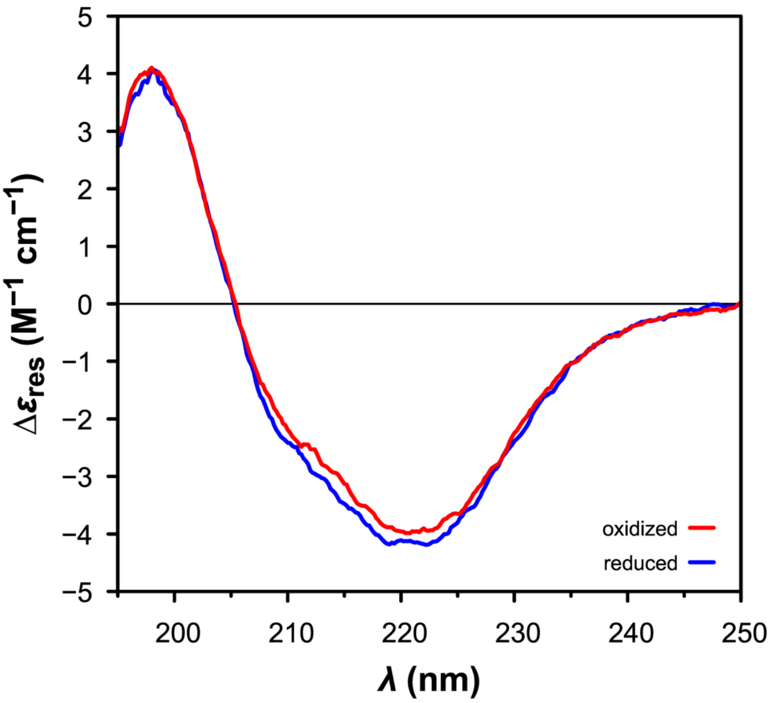

Figure S2.

Figure S3

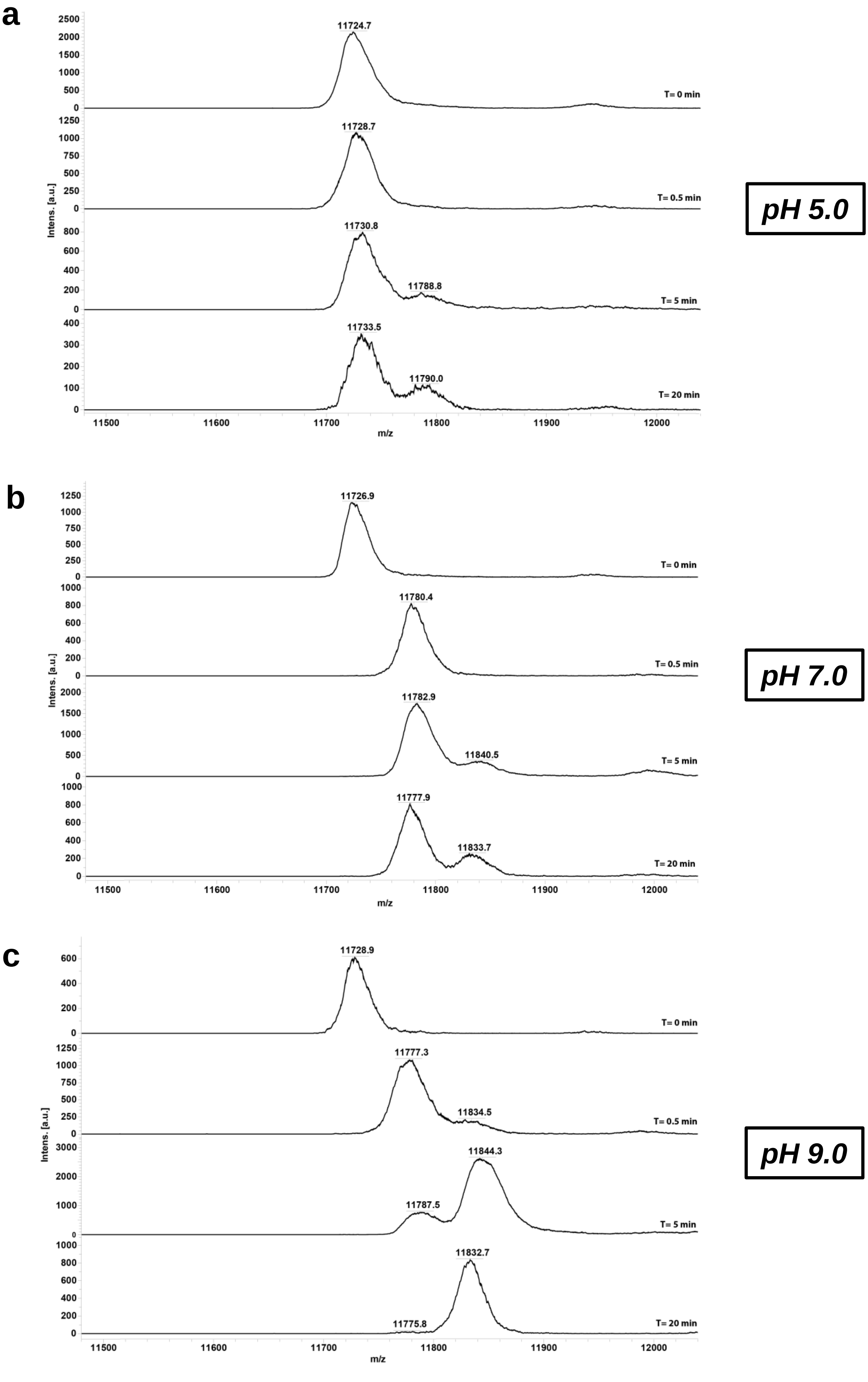
a
11724.7
T= 0 min
11728.7
T= 0.5 min
11730.8
11788.8
T= 5 min
11733.5
11790.0
T= 20 min
Intens. [a.u.]
m/z
pH 5.0
b
11726.9
T= 0 min
11780.4
T= 0.5 min
11782.9
11840.5
T= 5 min
11777.9
11833.7
T= 20 min
Intens. [a.u.]
m/z
pH 7.0
c
11728.9
T= 0 min
11777.3
11834.5
T= 0.5 min
11844.3
11787.5
T= 5 min
11832.7
11775.8
T= 20 min
Intens. [a.u.]
m/z
pH 9.0

Figure S4

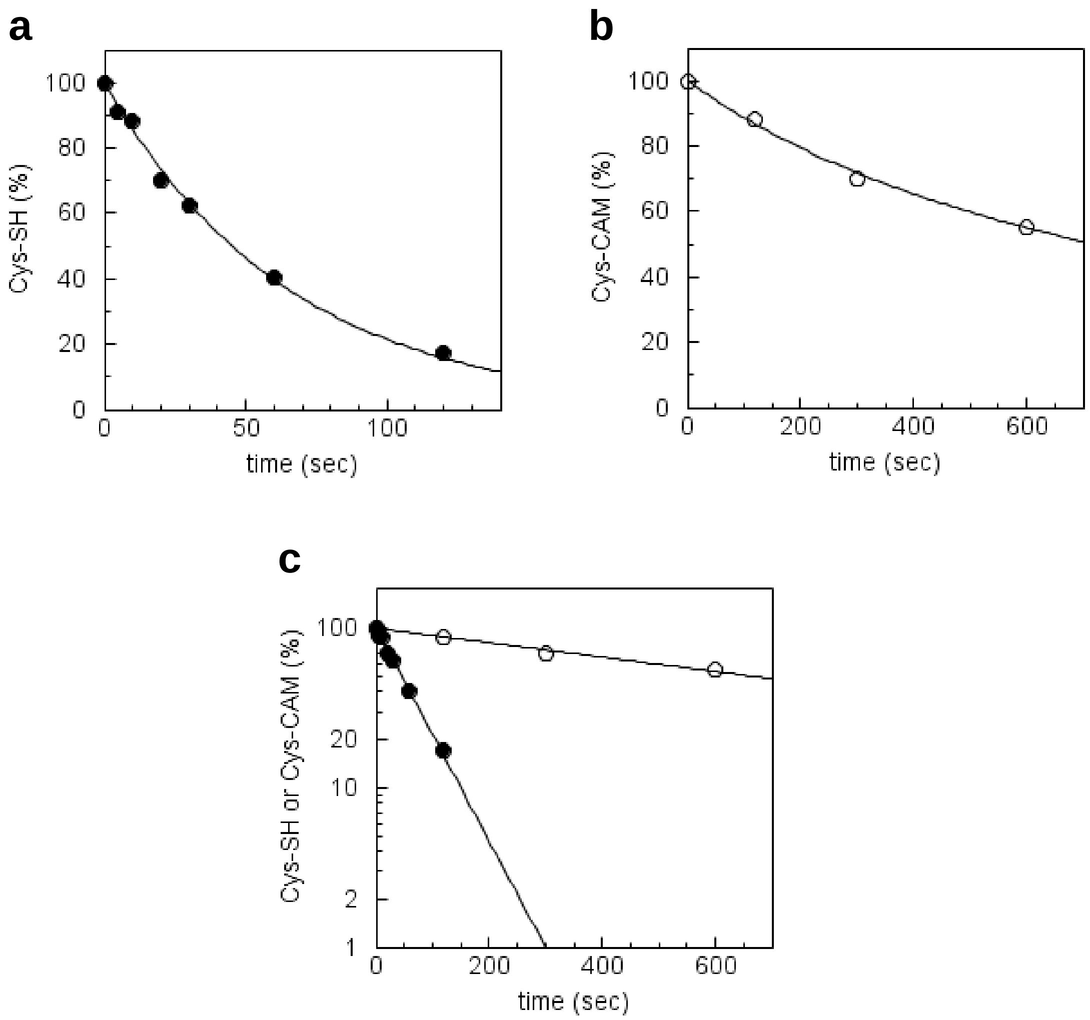
a
100
80
60
40
20
0
Cys-SH (%)
0
50
100
time (sec)
b
100
80
60
40
20
0
Cys-CAM (%)
0
200
400
600
time (sec)
c
100
20
10
2
1
Cys-SH or Cys-CAM (%)
0
200
400
600
time (sec)

Table S1

Chains		Distance (Å)	
A (atom)	B (atom)	Oxidized	Reduced
Lys40 (NZ)	Glu72 (OE1)	3.5	3.4
Glu72 (OE1)	Lys40 (NZ)	2.5	/
Met79 (N)	Thr77 (O)	2.8	3.0
Thr77 (O)	Met79 (N)	2.9	2.9
Trp35 (NE1)	Val64 (O)	3.6	3.4
Trp35 (NE1)	Asp65 (OD1)	2.9	3.2
Val64 (O)	Trp35 (NE1)	4.0	4.0
Asp65 (OD1)	Trp35 (NE1)	/	4.0
